# Implementation science in nutrition practice: A review of the Consolidated Framework for Implementation Research

**DOI:** 10.1002/ncp.70020

**Published:** 2025-08-30

**Authors:** Bridve Sivakumar, Jordan Mak, Salma Bafagih, JoAnne Arcand

**Affiliations:** ^1^ Faculty of Health Science Ontario Tech University Oshawa Ontario Canada; ^2^ Toronto General Hospital University Health Network Toronto Ontario Canada

**Keywords:** consolidated framework for implementation science, diet, dietary interventions, implementation science, nutrition

## Abstract

Many nutrition interventions and innovations are supported by strong evidence; however, their adoption, implementation, and long‐term sustainability in real‐world healthcare settings too frequently remain a challenge. Implementation science offers methodologies to equip practitioners with tools to identify and address the contextual factors that influence implementation success in health settings (e.g., adoption, implementation, sustainability). Among the various frameworks and theories used in implementation science, the Consolidated Framework of Implementation Research (CFIR) is one of the most widely used. The CFIR synthesizes constructs from multiple behavioral and implementation theories into a comprehensive tool that can be used to systematically assess the barriers and facilitators that influence implementation outcomes. The framework enables practitioners and researchers to identify context‐specific implementation determinants and to design tailored implementation strategies across diverse contexts and settings. Given its adaptability, the CFIR is highly relevant to the field of nutrition and dietetics to support sustained adoption and delivery of nutrition innovations (e.g., screening tools, educational programs, quality improvement initiatives); but it is relatively underutilized in nutrition practice. This article provides an overview of the CFIR and illustrates how it can be used to guide the implementation of nutrition innovations in clinical practice through two pragmatic case studies. We highlight the potential of the CFIR to be used as a guiding framework to advance the adoption, implementation, and sustainability of nutrition innovations and improve nutrition care and patient outcomes.

## INTRODUCTION

Sustaining long‐term adoption and delivery of new innovations in healthcare settings is a widely recognized challenge.^1^, ^2^ Despite strong evidence of their impact, many innovations fail to achieve meaningful integration into practice or yield beneficial improvements in health‐related outcomes.^1^ In fact, studies estimate that only 23% of interventions are sustained 2 years after implementation.^1^ As a result, there is a growing interest in the application of implementation science research methodologies in clinical practice.^1^, ^2^ Implementation science theories and frameworks support the effective uptake and sustained impact of evidence‐based health innovations in healthcare and other organizational settings.^2^ These include process frameworks (e.g., Knowledge‐to‐Action framework^3^), which guide the systematic process of knowledge creation to its translation and application in practice settings, and classical theories (e.g., social cognitive theory^4^), which describe mechanisms of behavior change and inform the design of innovations and implementation strategies. This consideration includes nutrition innovations, which can be described as a range of interventions, programs, and services designed to improve nutrition care and/or the nutrition status of individuals or populations, such as screening tools, educational programs, novel interventions, or other quality improvement initiatives.

The practical use of implementation science principles can help clinicians identify and evaluate the contextual factors that influence implementation outcomes to improve innovation adoption and delivery. Determinant frameworks help identify the contextual factors, conditions, and mechanisms that act as barriers or facilitators to implementation, which in turn impact key outcomes such as adoption, implementation, and sustainability. By applying a determinant framework, implementers can proactively address implementation challenges and enhance the effectiveness and long‐term sustainability of both innovations and implementation strategies.

One of the most widely cited^5^, ^6^ determinant frameworks is the Consolidated Framework of Implementation Research (CFIR),^7^ which was established to overcome a persistent challenge in implementation science: the lack of an existing comprehensive theory or framework that captures the breadth of factors that influence implementation success. Earlier theories and frameworks offered useful constructs for understanding implementation, but each was limited when used in isolation, capturing some implementation factors while overlooking others. No single theory or framework provided a comprehensive view of factors that influence implementation. The CFIR addressed this limitation by amalgamating the constructs from numerous behavioral and implementation science theories and frameworks into a unified, meta‐theoretical framework, enabling a holistic evaluation of implementation. When systematically applied, the CFIR assists implementation teams in identifying and assessing (diagnosing) relevant context‐specific factors that serve as barriers and enablers to implementation. This knowledge can equip teams in taking an evidence‐informed approach to adapting innovations, tailoring implementation strategies, and assessing progress, thereby improving implementation outcomes and enhancing the overall performance and effectiveness of an innovation.

The CFIR is pragmatic, and there is an opportunity to expand its use within the field of nutrition and dietetics. This is particularly important in the clinical setting, in which implementing nutrition interventions and programs can be especially challenging because of factors such as limited resources, lack of management support, staff turnover, and lack of interdisciplinary collaboration.^8^ Therefore, in this article we (A) describe the CFIR as a tool to support the implementation of evidence‐based interventions in health and other organizational settings and (B) outline how the application of the CFIR can enhance the implementation of clinical nutrition interventions in inpatient and outpatient settings using two case studies as illustrative examples.

## OVERVIEW OF THE CFIR

### Development of the CFIR domains and constructs

The CFIR was first introduced by Damschroder et al.^7^ Its development involved a snowball sampling approach in which 19 widely cited theories from health services research were identified and systematically examined. The constructs from these theories were evaluated by implementation science researchers considering their hypothesized influence on implementation outcomes; overlapping constructs were merged, whereas conceptually distinct constructs were retained as separate entities. This process resulted in a framework of standardized taxonomy, terminology, and definition of determinants that influence implementation success to help understand how and why implementation efforts succeed or fail in real‐world settings. In 2022, the CFIR was updated based on feedback from researchers who had applied the tool in diverse real‐world contexts.^9^

**Figure 1:**
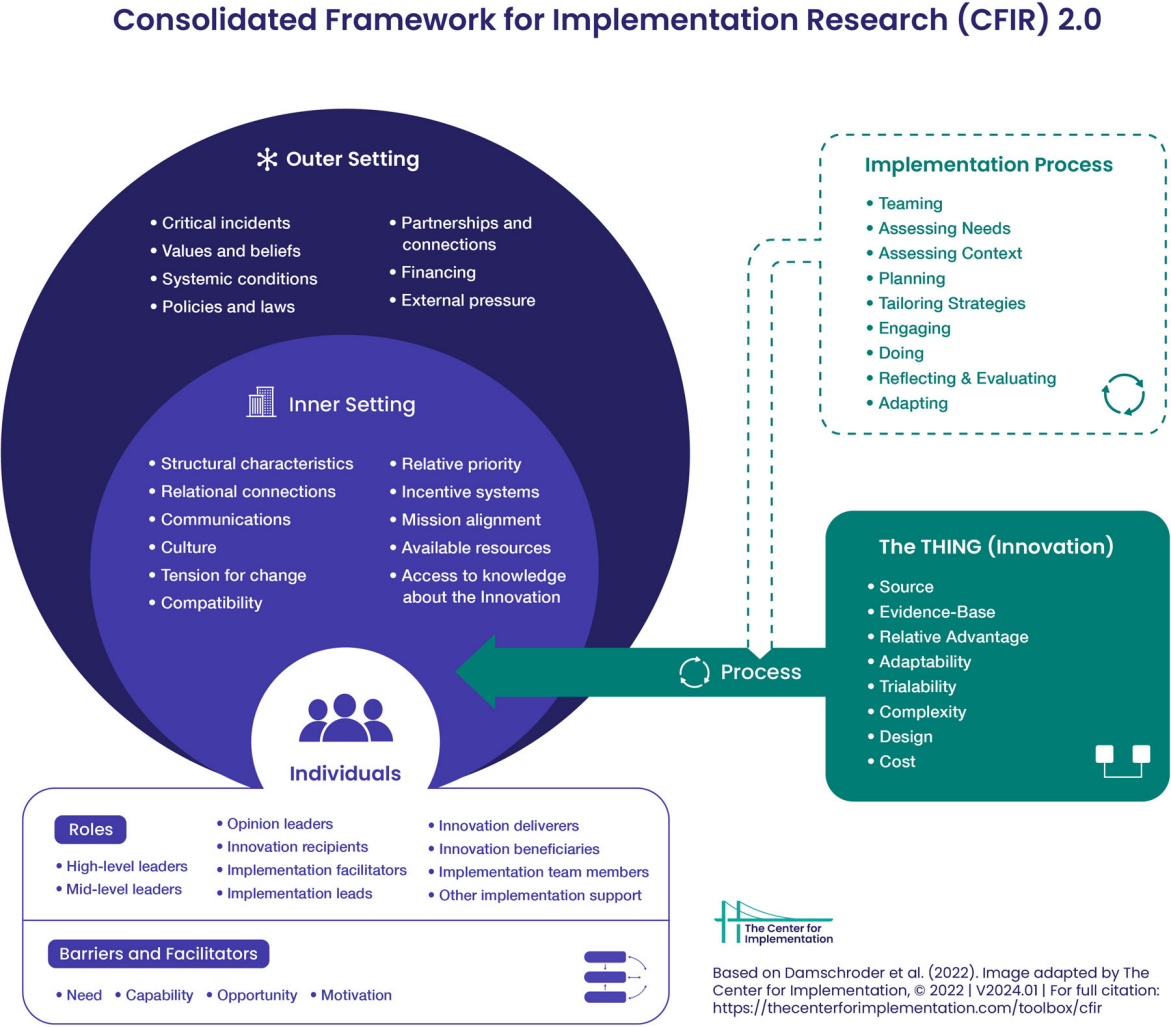
The Consolidated Framework of Implementation Research. CFIR 2.0. Adapted from Damschroder et al.^10^ The updated consolidated framework for implementation research based on user feedback. *Implementation Science, 17*, 75. https://doi.org/10.1186/s13012-022-01245-0. Image adapted by The Center for Implementation, 2022. Version: V2024.01. https://thecenterforimplementation.com/toolbox/cfir.

Figure 1 depicts the updated CFIR framework.^10^ The CFIR is organized into five main domains that influence implementation outcomes: (1) innovation characteristics: the new treatment, tool, educational program or service being implemented; (2) outer setting: the broader environment that influences the inner setting; (3) inner setting: the place where the innovation is being implemented; (4) individuals domain; the roles and characteristics of the individuals involved in the innovation; and (5) implementation process: the activities and strategies used to implement the innovation. In total, the CFIR includes 39 constructs, or determinants, across these five domains.

### Application of the CFIR

Through deductive reasoning, implementation teams can map the predefined CFIR constructs that exist across the five CFIR domains (e.g., innovation characteristics, outer setting, inner setting, individual's characteristics, and process) to their specific innovation and organizational context (setting). As a result, the systematic application of the CFIR enables implementers to generate “mechanistic” insights into why an outcome was achieved (or not), guided by the constructs (determinants). In other words, it can explain why and how implementation efforts succeed or fail within a given context. The implementation outcomes that teams typically strive to achieve include adoption (i.e., the decision to use the innovation), implementation (the extent to which the innovation will be, or was, delivered), and sustainability (i.e., the extent to which the innovation will be, or was, delivered over the long‐term).^11^

**Figure 2:**
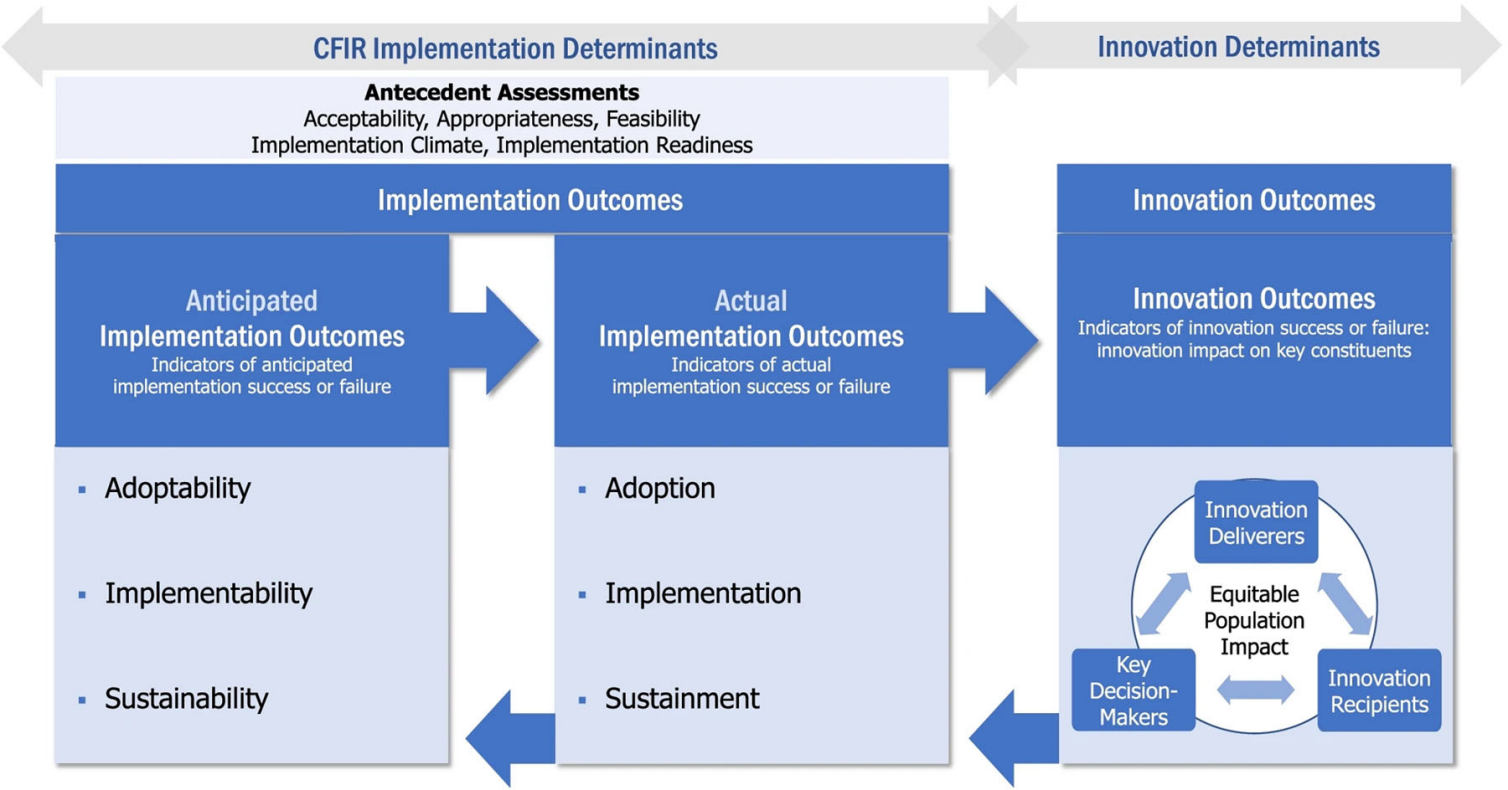
A contextual overview of implementation and innovation outcomes. Diagram of the CFIR Outcomes Addendum. From: Damschroder et al,^10^ licensed under CC BY 4.0 (https://creativecommons.org/licenses/by/4.0/).

The CFIR framework can be applied at all stages of the implementation process to guide decision‐making to maximize implementation of an innovation. Implementation determinants can be assessed relative to anticipated determinants (during preimplementation assessments) or actual determinants (during or postimplementation assessments) that have led to, or are expected to lead to, implementation success or failure, relative to implementation outcomes (Figure 2).^11^

- In the preimplementation phase, the CFIR facilitates a systematic assessment of the anticipated contextual factors that may act as barriers or facilitators to achieving implementation outcomes, such as organizational readiness, external environmental forces, or training needs. The use of the CFIR during preimplementation supports implementation teams in developing and tailoring implementation strategies to a specific organizational context to minimize barriers and leverage facilitators.
- During implementation, the CFIR provides a framework for monitoring how assessed (actual) determinants are positively or adversely influencing implementation efforts; ideally enabling timely adjustments in rapid iterative succession to enhance implementation success. Using the CFIR during this phase of implementation allows implementation teams to refine and optimize their implementation strategies.
- In the postimplementation phase, the CFIR can guide an analysis of which (actual) factors supported or hindered the implementation process and the success or failure in achieving implementation outcomes. Insights from this phase can guide how the innovation can be sustained, scaled, or refined in the future.

The CFIR framework is designed to be flexible and adaptable, allowing users to selectively apply the constructs that are most relevant to their unique setting, context, and innovation. In other words, not every construct needs to be used with each application of the CFIR; rather, the framework supports the selective use of constructs based on the needs and conditions of the implementation context.

Additionally, the metrics evaluated can be a combination of formal and/or informal qualitative and quantitative assessments, often obtained from multiple stakeholders involved in the implementation processes. The CFIR can be used in tandem with other evaluation frameworks used in implementation science (e.g., Reach, Effectiveness, Adoption, Implementation, and Maintenance [RE‐AIM] framework^12^, ^13^). Unlike evaluation frameworks, which are used to assess the impact and effectiveness of implementation strategies, the CFIR is designed to support the identification and assessment of determinants that shape implementation outcomes. Its complementary nature allows for a deeper understanding and context of the factors that led to adoption, implementation, and sustainability of the innovation in practice. Achieving implementation outcomes is essential for the innovation to achieve its intended impact. Indeed, a poorly implemented nutrition innovation is unlikely to achieve its intended effect on nutrition or health‐related outcomes. It should be noted that examining actual implementation outcomes is different from determining outcomes associated with the innovation itself (innovation outcomes), although the two are closely linked, as articulated by the CFIR Addendum published in 2022 (Figure 2).^11^ Strong implementation outcomes greatly influence the success of the innovation outcomes themselves since innovation deliverers, innovation recipients, and decision makers can influence intervention effectiveness and intended impact.

### The use of the CFIR in nutrition and dietetics

A literature search conducted in writing this narrative review revealed that the CFIR framework has been used to inform the implementation of nutrition innovations in a variety of settings, such as hospitals, primary care, long‐term facilities, and in low‐ and high‐resource communities, with studies applying the framework at different phases of the implementation process.^14^, ^15^, ^16^, ^17^, ^18^, ^19^, ^20^, ^21^

In the context of nutrition innovations, the CFIR has most commonly been used during the preimplementation phase. For instance, Taher et al^20^ used the CFIR to evaluate current practice and map constructs related to food insecurity screening and referral processes in primary care settings. Constructs from the CFIR informed the development of semistructured interview questions for healthcare staff. The CFIR was also used to code and analyze the qualitative interview data. Several barriers to implementation of food insecurity screening were identified, including limited internal resources to address food insecurity. Meanwhile, facilitators included a supportive implementation environment (e.g., perceptions and attitudes of staff and patients), and strong community partnerships. These insights on current practices related to food insecurity screening and referrals contributed to the development and testing of a conceptual model to inform future guidelines in primary care practice.

In another study, Beichmann et al^15^ used the CFIR to evaluate current nutrition support practices for patients with cancer at a university hospital in Norway and to identify barriers and factors that could support the implementation of a new nutrition intervention called Green Approach to Improved Nutrition (GAIN). Prior to implementing GAIN, individual interviews and focus groups were conducted with healthcare professionals and patients with cancer and then deductively coded and thematically analyzed using the CFIR. This study identified several barriers (e.g., limited technological knowledge, lack of patient motivation) and facilitators (e.g., desire for change), which helped plan the implementation of the GAIN intervention in a randomized controlled trial.

Although there are a limited number of research articles available, the CFIR has also been applied during the implementation phase for nutrition innovations. For instance, a mixed‐methods implementation evaluation used the CFIR to assess the implementation process during a pilot study of a perioperative nutrition care pathway in patients undergoing upper gastrointestinal cancer surgery.^17^ Focus groups and quantitative surveys were conducted with dietitians, patients, and multidisciplinary team members over the course of approximately 1 year; and qualitative data were thematically analyzed using CFIR. Adaptations were made iteratively during the pilot study in response to implementation challenges identified through the evaluation, such as compatibility of the nutrition care pathway with existing care. Several benefits to the implementation of the care pathway were also identified, such as increased engagement between dietitians and multidisciplinary team members and a proactive approach to nutrition care. The findings contributed valuable insight into how the implementation of the perioperative nutrition care pathway could be further improved to support broader implementation efforts.

The CFIR has also been instrumental in the postimplementation phase for nutrition innovations. For example, Endris et al^18^ used the CFIR to assess factors that influenced implementation of nutrition innovations that focused on growth monitoring, iron–folic acid supplementation, and nutrition counseling in primary care health settings in Ethiopia. Postintervention interviews with stakeholders and service providers were guided by the CFIR constructs. The interview transcripts were also deductively analyzed using the framework. The authors identified several factors that facilitated implementation such as adaptability and tension for change, as well as barriers, including poor staff commitment and motivation, false reporting, poor knowledge among service providers, and lack of engagement from leaders. These insights were crucial in identifying several strategic actions needed to support implementation success in the broader Ethiopian healthcare context, including the need for separate growth monitoring rooms and healthcare provider training.

In another study, Coffey et al^16^ applied the framework at both preimplementation and postimplementation timepoints to identify and explore the factors that influence the implementation of three evidence‐based guidance documents on managing pain, medication, or hydration and nutrition in three long‐term care facilities. A situational analysis was conducted using the CFIR framework, along with the Promoting Action on Research in Health Services (PARiHS) framework,^16^, ^22^ as part of preimplementation to gain understanding of the context at the three study sites. During the preimplementation phase, data were collected through staff surveys to assess learning needs, practice audits, and work‐based learning groups that discussed the practice changes required for implementation of the guidance documents. Postimplementation data included a repeat of the situational analysis, as well as interviews with champions to assess their perspectives on the process and effectiveness of the implementation. The CFIR was used as a coding template for the qualitative interviews conducted both preimplementation and postimplementation with both quantitative and qualitative findings presented in accordance with the CFIR framework. This study emphasizes the usefulness of the CFIR framework applied at preimplementation to identify factors to help guide intervention implementation and at postimplementation to evaluate outcomes and processes to make recommendations for practice.

Collectively, these studies^15^, ^16^, ^17^, ^18^, ^20^ demonstrate the comprehensiveness of the CFIR and its application to determine the wide range of contextual factors that influence implementation of nutrition interventions and services to improve practice.

### Understanding the CFIR domains: Framework overview and case study application

To illustrate how the CFIR can be applied to maximize the implementation of nutrition innovations in nutrition practice, we have applied it to two hypothetical case studies: one case focusing on the implementation of a nutrition screening tool in an inpatient hospital setting and one centered on the application of a new counseling approach in an outpatient primary care setting. These scenarios are briefly described below and then used as examples in the rest of this review as to how the CFIR framework can be applied to comprehensively assess implementation barriers and enablers that may be encountered and how these can be addressed or leveraged as part of a broader implementation strategy.

**Inpatient case study:** A dietitian practices in a high‐acuity inpatient unit at an academic hospital in Canada. Malnutrition in this population is common, and it is estimated that malnourished patients stay 3 days longer in hospital compared with those without malnutrition.^32^ To identify patients at nutrition risk, the dietitian successfully piloted a nutrition screening initiative in a single surgical unit using the Canadian Nutrition Screening Tool (CNST). On patient admission to the unit, nurses were trained to use and document the two‐question screening tool. The implementation of the tool was a success, as measured by the number of patients who received nutrition screening, were diagnosed with malnutrition, and received nutrition intervention. This program has encouraged the dietitians’ manager to seek expansion of the screening to six additional inpatient medical and surgical units in which no systematic nutrition screening is currently taking place. To successfully lead implementation and sustainability of the program across additional units, the dietitian uses the CFIR to evaluate which factors supported the implementation of the CNST on the pilot unit and to assess factors that may influence the expansion to additional hospital units.

**Outpatient case study:** A dietitian practices in a multidisciplinary primary care clinic in Ontario, Canada. The dietitian has traditionally followed weight‐centric approaches, emphasizing weight loss as a key strategy for improving health outcomes. However, patients routinely express frustration with weight‐focused dietary interventions as they have not led to sustained weight change or long‐term improvements in health. Some patients even avoid seeking medical care because of past experiences of weight stigma in healthcare settings. This shift in practice would align with clinic‐based initiatives focused on making the space more accessible and acceptable for people with larger body sizes. After attending webinars and reviewing new research, the dietitian has become interested in shifting her practice toward a weight‐inclusive approach, which focuses on health behaviors rather than weight loss as a primary outcome. The dietitian decides to adopt a weight‐inclusive model rooted in Health at Every Size (HAES) that promotes body respect, intuitive eating, and joyful movement, rather than focusing on weight loss as a measure of success. The dietitian uses the CFIR to understand the barriers and enablers she needs to consider to ensure implementation success.

## INNOVATION CHARACTERISTICS DOMAIN

The innovation characteristics domain of the CFIR focuses on the specific features of an innovation that may influence implementation and adoption within a particular setting, such as a healthcare organization (Table 1). This domain helps implementation teams assess how well a new innovation, such as a novel screening tool, a nutrition intervention, a digital innovation, or a new model of care delivery, fits within the existing practice environment. Questions implementers may ask include: How credible is the innovation? Is it adaptable to my organization or local context? Is it considered feasible and acceptable to those delivering or receiving it? Many nutrition innovations implemented in healthcare settings are inherently complex and multifaceted, as they often have multiple components and address complex health or care delivery issues. Thus, the implementation of a single nutrition innovation can vary greatly across different organizational settings, requiring an array of adaptations, resources (such as costs and training), as well as workflow and practice changes. The constructs within this domain, such as innovation relative advantage, innovation adaptability, innovation complexity, innovation trialability, and innovation cost, allow implementers to closely examine the factors inherent to an innovation itself that may serve as a barrier or enabler to implementation success (adoption, implementation, sustainability), relative to the setting in which the innovation will be implemented. Understanding these factors is essential for designing effective implementation strategies and processes. For example, if a nutrition innovation is perceived by innovation deliverers as being burdensome, unadaptable, or costly, it will be less likely to be adopted regardless of its evidence base.^23^, ^24^ In contrast, a nutrition innovation that aligns well with existing clinical practices, that is supported by strong evidence, and that has low complexity will be more likely to be accepted and adopted by innovation deliverers. By examining the characteristics of a nutrition innovation that may help or hinder implementation efforts, implementation processes can be tailored to consider any anticipated challenges, make necessary adaptations to the innovation, or consider alterations of processes to ensure successful implementation and adoption.

**Table 1:** CFIR innovation characteristics domain constructs.

Construct	Description* The degree to which:	Example implementation questions related to nutrition innovations
**Innovation source**	The group that developed and/or visibly sponsored use of the innovation is reputable, credible, and/or trustable.	Who developed or sponsored the nutrition innovation (e.g., a local health agency, university, dietitian, physician)? Do stakeholders trust the source behind the nutrition innovation? When an external source developed the nutrition innovation, was there transparency in the decision‐making process to adopt the source within the organization?
**Innovation evidence‐base**	The innovation has robust evidence supporting its effectiveness.	What evidence supports the nutrition innovation's impact on its intended outcomes (e.g., dietary behavior detection of malnutrition)? Has it been tested in similar settings and populations? Are the results applicable to our setting?
**Innovation relative advantage**	The innovation is better than other available innovations or current practice.	How does this nutrition innovation compare to what we already do and/or to similar innovations? What makes it more beneficial? Do all staff or other stakeholders perceive it as a clear improvement to current practice?
**Innovation adaptability**	The innovation can be modified, tailored, or refined to fit local context or needs.	Is the nutrition innovation flexible for various settings, intervention deliverers or intervention recipients? Does it need to be refined to be implemented into the practice setting (e.g., considering cultural dietary patterns, language needs, literacy levels)? Can the nutrition innovation be adapted and still stay true to its intended purpose?
**Innovation trialability**	The innovation can be tested or piloted on a small scale and undone.	Can the implementation of a nutrition innovation be pilot tested in one site (e.g., one primary care clinic, one unit) prior to broad implementation? Can a nutrition innovation be stopped or adjusted easily, if needed? Can feedback be collected during the trial?
**Innovation complexity**	The innovation is complicated, which may be reflected by its scope and/or the nature and number of connections and steps.	How many steps or stakeholders are involved in implementation? Are materials or instructions overly complex? Is intensive training or coordination required to deliver the nutrition innovation?
**Innovation design**	The innovation is well designed and packaged, including how it is assembled, bundled, and presented.	Is the nutrition innovation easy to understand and engaging? Is the overall structure (e.g., lesson flow, visuals) clear? Does the design make it accessible and easy to implement?
**Innovation cost**	The innovation purchase and operating costs are affordable.	Are costs (e.g., food, printing, training) manageable within our budget? Can it be sustained long‐term?

CFIR, Consolidated Framework of Implementation Research.

* Description from: Damschroder et al.,^9^ licensed under CC BY 4.0 (https://creativecommons.org/licenses/by/4.0/).

### Inpatient case

The CNST is a validated two‐question screening tool used to systematically identify patients at risk for malnutrition who may benefit from further assessment. The tool has high trustworthiness as it was developed by nutrition professionals and researchers as part of the Canadian Malnutrition Task Force, a leading standing committee on the topic within the Canadian Nutrition Society (innovation source). The CNST is evidence‐based, with research showing high reliability, specificity, and sensitivity in the detection of malnutrition; and good predictive validity for length of stay, 30‐day hospital readmission, and mortality (innovation evidence base*)*.^25^ The CNST is not a complex intervention to implement, as it includes only two standardized questions that can quickly and easily be administered by most members of the healthcare team (innovation complexity). Its simplicity enhances the feasibility and scalability of implementation across inpatient settings, facilitating early malnutrition risk detection. It is concise and can be easily integrated into electronic health records as part of the nursing admission assessment with minimal training, and is available free of charge (innovation design, innovation cost). The brevity of the CNST poses a clear relative advantage over tools such as the Malnutrition Screening Tool,^26^ which requires quantifying weight loss, or the Mini Nutritional Assessment ‐ Short Form,^27^ a longer 6‐question tool. Although the CNST cannot be modified or tailored to specific contexts, this does not pose a barrier when applied within the inpatient setting (innovation adaptability). The CNST can feasibly be tested on a smaller scale as demonstrated by the pilot on the dietitian's surgical unit before broader implementation (innovation trialability). After review of the constructs within the innovation domain, the dietitian determines that the characteristics of the CNST largely supported the success of pilot implementation and that it remains the best tool to support expansion of nutrition screening across additional hospital units.

### Outpatient case

The dietitian reviewed the scientific literature on weight‐inclusive approaches and HAES, finding that these approaches are well‐supported by evidence and included in clinical practice guidelines (innovation evidence base).^28^ For instance, a recent meta‐analysis of intervention studies showed that individuals randomized to a HAES‐based intervention had significantly lower susceptibility to hunger, compared with those in weight‐centric control groups that emphasized restrictive dieting, with no significant difference between HAES and control groups for anthropometric, psychological or cardiometabolic outcomes.^29^ Additionally, compared with weight‐centric approaches, HAES offers a clear relative advantage by reducing external and internalized weight stigma, enabling greater patient engagement and promoting long‐term sustainable health behaviors compared with weight‐centric approaches.^30^ The dietitian will be the primary deliverer of weight‐inclusive care, supported more generally by members of the healthcare team. However, the dietitian identifies potential challenges to implementation success after review of innovation characteristics: weight‐inclusive care is characterized by high innovation complexity as it requires tailoring to individual patient needs (i.e., nonstandardized), consideration of the social determinants of health, and the integration of psychological support and strategies to address systemic barriers. Although its adaptability allows for modifications to consider the need for health‐specific medical nutrition therapy (e.g., diabetes, cardiovascular disease), weight‐inclusive care will require the dietitian to develop new skills in intuitive eating, trauma‐informed care, and harm reduction counseling strategies and to consider equity‐seeking adult clients’ experiences of oppression (innovation adaptability). The dietitian will also need to account for implementation‐related time and financial costs for professional development, including education for the multidisciplinary team, patient resource creation, and potential clinic policy updates (innovation costs).

## OUTER SETTING DOMAIN

The outer setting domain of the CFIR assesses the forces outside an organization (or other inner setting) that can shape implementation efforts, such as economic, political, social, and community‐level “macrolevel” factors (Table 2). The outer setting constructs domain captures factors such as government policies, funding structures, clinical or public health priorities, and broader societal attitudes or norms. For example, changing national clinical or public health priorities can influence the degree of success an organization has in adopting, integrating, and sustaining the implementation of a new nutrition innovation. Assessing the outer setting allows for an understanding of contextual barriers and enablers that may be outside an organization's immediate control but are critical to implementation success. Identifying these factors enables a broader awareness and allows implementers to tailor internal implementation strategies relative to external dynamics.

**Table 2:** CFIR outer setting domain constructs.

Construct	Description* The degree to which:	Example implementation questions related to nutrition innovations
**Critical incidents**	Large‐scale and/or unanticipated events disrupt implementation and/or delivery of the innovation.	Has a recent crisis or event (e.g., pandemic, food shortage, natural disaster) affected community nutritionaneeds and priorities? Have these events impacted our ability to deliver nutrition services (e.g., changes in government mandates, health system directives)?
**Local attitudes**	Sociocultural values (e.g., shared responsibility in helping recipients) and beliefs (e.g., convictions about the worthiness of recipients) encourage the Outer Setting to support implementation and/or delivery of the innovation.	What are the values, norms, and beliefs of the community or external environment (e.g., geographic area, cultural setting, professional field) that may support or hinder implementation? How do these attitudes influence how the nutrition innovation is perceived and received by external stakeholders (e.g., patients, decision makers, professional networks)?
**Local conditions**	Economic, environmental, political, and/or technological conditions enable the Outer Setting to support implementation and/or delivery of the innovation.	How do the economic, political, environmental, technological, or geographic conditions within the outer setting influence external organizations (e.g., government, nongovernmental organizations) to support implementation or delivery of the nutrition innovation within the inner setting?
**Partnerships & connections**	The Inner Setting is networked with external entities, including referral networks, academic affiliations, and professional organization networks.	How connected is the implementation team with external organizations related to nutrition and/or the innovation of interest? Which organizations external to the team provide practical resources or in‐kind support to facilitate the implementation of the nutrition innovation, if needed? Are there potential partners (e.g., extension programs, nonprofits) that can help expand the reach or credibility of the nutrition innovation?
**Policies & laws**	Legislation, regulations, professional group guidelines and recommendations, or accreditation standards support implementation and/or delivery of the innovation.	What nutrition‐related policies, regulations, or guidelines at the local, state/provincial, federal, or international level can support the implementation of the nutrition innovation? How do existing mandates (e.g., hospital requirements for staff and visitors to wear masks during respiratory virus seasons) support or hinder the delivery of the nutrition innovation?
**Financing**	Funding from external entities (e.g., grants, reimbursement) is available to implement and/or deliver the innovation.	Are there grants or reimbursements available for implementation of the nutrition innovation? Is the funding received for the nutrition innovation sufficient and/or sustainable for long‐term implementation?
**External pressure**	External pressures drive implementation and/or delivery of the innovation. *This construct captures External Pressures that are not included in the subconstructs below*.	
A. Societal pressure	Mass media campaigns, advocacy groups, or social movements or protests drive implementation and/or delivery of the innovation.	Are there public health campaigns that align with the nutrition innovation? Are social movements (e.g., social justice, food sovereignty, food sustainability, health equity) influencing the priorities of individuals and groups outside the inner setting?
B. Market pressure	Competing with and/or imitating peer entities drives implementation and/or delivery of the innovation.	Have other organizations implemented similar, or the same, nutrition innovation? Are there feelings of pressure to keep up with others or to outshine competitors?
C. Performance‐ measurement pressure	Quality or benchmarking metrics or established service goals drive implementation and/or delivery of the innovation.	Are governments or funders requiring data on the nutrition innovation's performance or impact on patient/client outcomes, such as improved BMI, time to malnutrition diagnosis and treatment, glycemic control, patient satisfaction, etc?

Abbreviations: BMI, body mass index; CFIR, Consolidated Framework of Implementation Research.

* Description from: Damschroder et al.,^9^ licensed under CC BY 4.0 (https://creativecommons.org/licenses/by/4.0/).

### Inpatient case

Through a review of the outer setting, the dietitian can identify factors outside the institution that impact the ability to successfully implement the CNST. Over the last decade, a growing body of Canadian research has highlighted the adverse effects of malnutrition on patients, creating a favorable environment that supports the practice of nutrition screening (local conditions). This includes research estimating the prevalence of malnutrition at 45% of inpatients in the Canadian context^31^ and analysis suggesting malnourished patients stay 3 days longer in hospital with increased healthcare expenditures by 30%–55% per admission in Canada.^32^ Dissemination of this research has shaped beliefs about the role of malnutrition and the need to address it among healthcare professionals (local attitudes), influenced by values such as stewardship and quality care. Although no federal or provincial policies currently mandate nutrition screening, new Canadian health standards were developed in 2021 for malnutrition prevention, detection, and treatment: a precursor for nutrition screening to become a required organizational practice for accreditation (policies and laws, external pressure, and performance measurement pressure).^33^ Implementation of malnutrition screening on hospital admission also aligns with reputable practice guidelines published by the American Society for Parenteral and Enteral Nutrition (ASPEN) and the European Society for Clinical Nutrition and Metabolism (policies and laws).^34^, ^35^, ^36^ The dietitian is connected to organizations such as the Canadian Malnutrition Task Force (CMTF) supporting healthcare professionals in their efforts to improve malnutrition detection (partnerships and connections).^37^ This includes government advocacy efforts, professional development activities and initiatives to increase awareness such as Malnutrition Awareness Week (external pressure—societal pressure). ASPEN and the CMTF further support healthcare institutions through toolkits for the implementation of nutrition screening along with networking opportunities to form a community of practice (partnerships and connections). The increasing recognition and adoption of the CNST has created pressure for institutions to adopt this practice, with a 2022 study showing 78% of Canadian hospitals use the tool in at least one unit (external pressure—market pressure).^38^ Characterizing the outer setting using the CFIR reassures the dietitian that the external environment supports expansion of nutrition screening. By leveraging the components of the outer setting identified using this framework, the dietitian can justify the timing and importance of the initiative to support implementation.

### Outpatient case

A key driver to adopting a weight‐inclusive approach to care is its growing support from the health and dietetic community. The 2020 Obesity Canada clinical practice guidelines included weight‐inclusive care as a recommended approach to medical nutrition therapy (policies and laws). Recent studies estimate that approximately 45% of dietitians exclusively use weight‐inclusive approaches in their practice, whereas 41% fluctuate between using weight‐inclusive and weight‐centric care (local attitudes, external pressure).^39^ The dietitian is connected to professional organizations, such as Dietitians of Canada and Association for Size Diversity and Health (partnerships and connections) that support weight‐inclusive care. There are also informal communities of practice of dietitians who practice weight‐inclusive care on social media, further reinforcing its acceptance and support for implementation (local attitudes).

However, the dietitian acknowledges the strong influence of external pressure, namely societal pressure (i.e., norms and practices), that favors smaller body sizes may be a barrier to implementation success. This pressure may be reinforced specifically by patient and healthcare provider expectations of weight‐loss counseling on referrals to dietetic services (local attitudes). Moreover, the dietitian may need to navigate barriers posed by a lack of awareness and external skepticism related to weight‐inclusive care, recognizing that it is not universally accepted by the broader health community (external pressure).^40^ Finally, the dietitian investigates whether or not the clinic funding is dependent on weight‐based success metrics that are evaluated as part of programmatic reviews and evaluations, which is common (performance measurement pressure).^41^ After assessing the outer setting using the CFIR, the dietitian recognizes many supportive external structures she can draw on to support implementation. She also recognizes that some external barriers, especially societal norms regarding weight loss and body size, will need to be considered in the implementation plan. The dietitian is able to confirm that the clinic's funding is not contingent on weight‐based metrics of success and that this would not pose a barrier to implementation.

## INNER SETTING DOMAIN

The inner setting domain of the CFIR assesses factors related to the specific environment in which the innovation is implemented, such as a hospital, a school, a clinic, or a city (Table 3). Importantly, there may be multiple inner settings relative to implementation and/or different levels within an inner setting (e.g., hospital administration, a hospital ward, hospital food services). In applying the CFIR constructs, the inner setting domain consists of the structural, political, and cultural forces inside an organization that shape implementation efforts. This internal environment plays a key role in how a new nutrition innovation is received and operationalized in an organization, influencing factors such as staff buy‐in, communication processes, decision‐making, and resource allocation.^24^ By assessing the inner setting, implementation strategies can be tailored to fit the local context, better supporting adoption, integration, and sustainability of the nutrition innovation in nutrition practice.

**Table 3:** CFIR inner setting domain constructs.

Construct	Description* The degree to which:	Example implementation questions related to nutrition innovations
The following constructs exist in the inner setting regardless of implementation and/or delivery of the innovation, i.e., they are persistent general characteristics of the inner setting.		
**Structural characteristics**	Infrastructure components support functional performance of the Inner Setting. *Use this construct to capture themes related to Structural Characteristics that are not included in the subconstructs below*.	
A. Physical infrastructure	Layout and configuration of space and other tangible material features support functional performance of the Inner Setting.	Is the layout of the available space configured in a way that is safe, accessible, and private enough to implement the nutrition innovation?
B. Information technology infrastructure	Technological systems for tele‐communication, electronic documentation, and data storage, management, reporting, and analysis support functional performance of the Inner Setting.	Where appropriate, is there sufficient hardware (e.g., computers, tablets), software (e.g., electronic health records, dietary assessment tools), and data storage (e.g., cloud‐based systems) that meet the requirements of the nutrition innovation? Can stakeholders (e.g., patients, clinicians) easily access and use the technology required for the nutrition innovation?
C. Work infrastructure	Organization of tasks and responsibilities within and between individuals and teams, and general staffing levels, support functional performance of the Inner Setting.	How will the overall organization of staff member tasks and responsibilities impact implementation of the nutrition innovation? How will any required changes in staff workflow impact implementation?
**Relational connections**	There are high quality formal and informal relationships, networks, and teams within and across Inner Setting boundaries (e.g., structural, professional).	Are there strong working relationships between stakeholders at all relevant levels within the organization that may be impacted by implementation of the nutrition innovation (e.g., hospital units, diagnostic services, food services, across different types of healthcare professionals, etc)? Are there any barriers/facilitators for collaboration between stakeholders?
**Communications**	There are high quality formal and informal information sharing practices within and across Inner Setting boundaries (e.g., structural, professional).	What formal (e.g., email, staff meetings) and informal (e.g., friendly unscheduled interactions) communication channels can be used to support implementation efforts and at what frequency? How can stakeholders communicate and provide feedback to the implementation team?
**Culture**	There are shared values, beliefs, and norms across the Inner Setting. *Use this construct to capture themes related to Culture that are not included in the subconstructs below*.	
A. Human equality‐ centeredness	There are shared values, beliefs, and norms about the inherent equal worth and value of all human beings.	To what extent does the organization recognize and value the needs of diverse patient populations in nutrition care (e.g., culturally relevant diets, antistigma approaches) and how will this influence implementation of the nutrition innovation? How does the organization consider equity in decisions around access to nutrition care (e.g., referrals, nutrition screening, or counseling) and how could this impact implementation?
B. Recipient‐ centeredness	There are shared values, beliefs, and norms around caring, supporting, and addressing the needs and welfare of recipients.	To what extent does the organization make it a priority to tailor care to patient's needs and lived realities? Does the nutrition innovation consider how the innovation impacts patient food access, cultural practices, health literacy, etc?
C. Deliverer‐ centeredness	There are shared values, beliefs, and norms around caring, supporting, and addressing the needs and welfare of deliverers.	To what extent does the organization support staff in delivering nutrition care (e.g., adequate time, training, respect) and how can this impact implementation of the nutrition innovation? What are organizational values regarding nutrition care that could impact implementation?
D. Learning‐ centeredness	There are shared values, beliefs, and norms around psychological safety, continual improvement, and using data to inform practice.	Does the organizational culture enable a psychologically safe environment for stakeholders to learn and share feedback on the implementation of the nutrition innovation?
*The following constructs are specific to the implementation and/or delivery of the innovation.*		
**Tension for change**	The current situation is intolerable and needs to change.	To what extent do stakeholders view current nutrition practices as inadequate? Are there health outcomes or disparities that make change urgent (e.g., rise in foodborne illness, food insecurity, malnutrition, etc)?
**Compatibility**	The innovation fits with workflows, systems, and processes.	To what extent does the nutrition innovation align with existing health services? Can staff easily integrate the program into their daily routines and workflow?
**Relative priority**	Implementing and delivering the innovation is important compared to other initiatives.	How do stakeholders rank the importance of the nutrition innovation, relative to other health or nutrition innovations that would benefit the organization?
**Incentive systems**	Tangible and/or intangible incentives and rewards and/or disincentives and punishments support implementation and delivery of the innovation.	Are there rewards, such as financial incentives, job promotion, performance review, recognition and respect, for individuals involved in the implementation that support the nutrition innovation? Are there disincentives for nonparticipation?
**Mission alignment**	Implementing and delivering the innovation is in line with the overarching commitment, purpose, or goals in the Inner Setting.	To what extent does the nutrition innovation support the organization's overall mission, values, commitments, and strategic goals and objectives?
**Available resources**	Resources are available to implement and deliver the innovation. *Use this construct to capture themes related to Available Resources that are not included in the subconstructs below*.	
A. Funding	Funding is available to implement and deliver the innovation.	Are there budget constraints that could affect implementation? Are there opportunities for grants or external funding?
B. Space	Physical space is available to implement and deliver the innovation.	Does the organization have enough physical space to implement the nutrition innovation (e.g., private rooms, clinical assessment space, kitchen space)?
C. Materials & equipment	Supplies are available to implement and deliver the innovation.	Is equipment needed to implement the nutrition innovation (e.g., fridges, scales) available and functioning? What type of maintenance may be required?
**Access to knowledge & information**	Guidance and/or training is accessible to implement and deliver the innovation.	Is on‐the‐job training accessible to innovation deliverers so that they can effectively implement the nutrition innovation? Is ongoing support available to improve knowledge, skills, and confidence in delivery?

Abbreviation: CFIR, Consolidated Framework of Implementation Research.

* Description from: Damschroder et al.,^9^ licensed under CC BY 4.0 (https://creativecommons.org/licenses/by/4.0/).

### Inpatient case

The dietitian has demonstrated, through pilot implementation, that the institution currently has the *structural* characteristics in place to support broader implementation across the hospital. For instance, the screening questions have been incorporated into the electronic health record (structural characteristics—information technology infrastructure, compatibility). The surgical unit pilot also demonstrated that current staffing levels of nurses, nurse educators, and dietitians were sufficient to train and support nurses in achieving a high screening completion rate (structural characteristics—work infrastructure). Although the pilot surgical unit has the appropriate structural characteristics, the CFIR highlights the potential for the presence of multiple inner settings relative to implementation. In this case, each unit is a discrete inner setting. Therefore, the dietitian needs to not only explore the pilot unit setting but also identify differences in infrastructure, such as staffing levels, for each individual unit in which expansion of screening is implemented (structural characteristics—work infrastructure).

The presence of a long‐standing dietetic professional practice committee consisting of experienced dietitians at the hospital provides the peer network required to plan and execute new innovations (relational connections, communications). Additionally, a broader medical advisory committee composed of physicians, nurses, and hospital leaders who review changes to institutional policies form the structural and professional supports required to expand the CNST (relational connections, communications). Cultural forces within the inner setting are also highlighted by the dietitian as supporting the successful expansion of nutrition screening. The academic hospital's main values include stewardship, quality improvement, and innovation, which complement the implementation of best nutrition care practices (mission alignment). Changes required for the expansion of nutrition screening fit with the usual modifications of staff protocols because of the institution's high research activity and the frequent implementation of quality improvement initiatives, which form the norm around providing care within the hospital setting (culture—deliverer centeredness).

Through this assessment, the dietitian has an opportunity to further define the institution's readiness for change. For example, defining the relative priority of an expanded screening initiative will require collaboration with nurse managers and hospital leadership. The dietitian must be aligned with leaders on how nutrition screening practice is prioritized among competing clinical initiatives in a busy academic center. Furthermore, the dietitian must consider the available resources in the hospital. Despite being a low‐resource intervention to pilot on one inpatient unit, hospital‐wide implementation has greater implications on human resources, including nursing time and the ability for dietitians to assess patients who screen positive for risk of malnutrition (available resources: funding).

In an effort to better define available resources and relative priority, the dietitian professional practice committee reports the successes of the pilot project to hospital executives responsible for patient safety and quality. The committee advocates for expanded implementation of nutrition screening across units to improve malnutrition detection as a means to reduce costs and improve patient outcomes. The hospital executives are enthusiastic about the success of the pilot project and agree to support expansion of nutrition screening as a relative priority. However, they warn that limited financial resources are available. This helps the dietitian identify available resources as a potential barrier to implementation success, suggesting the possible need to apply for funded innovation fellowships or quality improvement grants if financial needs arise. Analysis of the inner setting highlights the institution's readiness for expansion of nutrition screening, taking into account the importance of cost‐neutral implementation and the additional steps that may be required if the need to secure funding arises.

### Outpatient case

A core organizational value of the clinic is a commitment to equity‐ and client‐centered care (culture: human equality centeredness, culture: recipient centeredness). With the goal of reducing barriers to healthcare, the adoption of weight‐inclusive care is in line with the clinic's values (mission alignment) and positions it as a relative priority within this context. The clinic's dedication to stigma‐free care helps to foster a culture of continual improvement and adopting new practices (culture: learning centeredness). This commitment is backed by a continuing education fund, which will help staff access the training required to implement weight‐inclusive care (available resources: funding, access to knowledge and information) and ensure the dietitian professional development funds for travel to training and mentorship programs.

Although the implementation of weight‐inclusive care will not impact clinic workflow (compatibility) or require changes in space requirements (available resources: space), the dietitian acknowledges changes to weight‐inclusive care impacts the entire team. Modifications to protocols and care algorithms would be needed, to ensure alignment with weight‐inclusive care, such as weight not being measured or considered as an outcome of care during routine physical examinations. Fortunately, the multidisciplinary nature of the clinic results in strong collaboration, communication, and rapport building among staff (relational connections). This new care model facilitates open discussions about practice change, which will help in improving the acceptability of weight‐inclusive care (communications). The clinic also offers opportunities for training and promoting team alignment, including monthly staff meetings, biweekly clinical workshops (access to knowledge and information), and informal interactions in shared spaces like the lunchroom that will offer opportunities for influence (communications*)*. The dietitian recognizes that the strong influence of external societal pressures and broader clinical and cultural norms about weight loss, may influence attitudes in the inner setting. Conflicting beliefs on weight‐inclusive care among both intervention deliverers (e.g., physicians, nurses) and intervention recipients (e.g., patients, caregivers) may pose a barrier to implementation (culture: deliverer centeredness, culture: recipient centeredness*)*. After assessing the inner setting environment, the dietitian recognizes that there are many factors that will enable her success, such as mission alignment, a strong collegial culture, easy integration into workflow, and access to resources for professional development. The dietitian recognizes that the traditional medical and cultural paradigms around weight loss may persist and impede implementation efforts.

## INDIVIDUALS DOMAIN

The individuals domain of the CFIR examines the roles and attributes of the individuals within an organization or setting (e.g., the inner setting) who are involved in implementing, delivering, or receiving the innovation (Table 4). This domain is crucial because organizational change is ultimately enacted by individuals, and the attitudes, abilities, and actions of individuals in an organization can significantly influence the success in adoption, implementation, and sustainability of a nutrition innovation. This domain captures two subdomains: (1) A roles subdomain identifies the various positions an individual holds within an organization that play a part in implementation success, such as leaders, facilitators, team members, deliverers, and recipients; (2) The characteristics subdomain focuses on individual‐level factors, such as skills, autonomy, and level of involvement in implementation, considering an individual's needs, capability, opportunity, and motivation as defined by the Capability, Opportunity, Motivation, and Behavior model.^42^ By evaluating the characteristics of individuals that may serve as barriers or enablers to implementation success, implementers can identify sources of support or tensions to change, pinpoint training and resource needs, and proactively and strategically engage individuals to build a culture that supports adoption, integration, and sustainability of a nutrition innovation in nutrition practice.

**Table 4:** CFIR individuals domain constructs.

Construct	Description* The degree to which:	Example implementation questions related to nutrition innovations
**Roles subdomain** Project roles: the roles of individuals involved with implementing, delivering, and/or receiving the innovation.		
**High‐level leaders**	Individuals with a high level of authority, including key decision‐makers, executive leaders, or directors.	How engaged and committed are high‐level leaders (e.g., executive leadership, administrators, decision makers) within the organization supporting the nutrition innovation, including allocating necessary resources to facilitate cultural change, structural adaptations, implementation sustainability, etc?
**Mid‐level Leaders**	Individuals with a moderate level of authority, including leaders supervised by a high‐level leader and who supervise others.	Are midlevel leaders in the organization clearly communicating expectations and aligning staff with the goals of the nutrition innovation? How are they supervising and supporting staff in implementation, and do they have the skills to do so?
**Opinion leaders**	Individuals with informal influence on the attitudes and behaviors of others.	Who are the informal influencers in the organization who can positively or negatively shape attitudes toward the nutrition innovation? What strategies and channels do opinion leaders use to assert their influence?
**Implementation facilitators**	Individuals with subject matter expertise who assist, coach, or support implementation.	Are there enough trained facilitators available to guide staff through implementation of the nutrition innovation? Do implementation facilitators have enough support and resources to fulfill their role (e.g., time, training materials)?
**Implementation leads**	Individuals who lead efforts to implement the innovation.	Who is taking primary responsibility for leading the nutrition innovation implementation, and what skill, time, and authority do they have to do so?
**Implementation team members**	Individuals who collaborate with and support the Implementation Leads to implement the innovation, ideally including Innovation Deliverers and Recipients.	Which team members, and how many, are needed to support the implementation of the nutrition innovation? Have the “right people in the right seats” been included on the implementation team? Is anyone missing?
**Other implementation support**	Individuals who support the Implementation Leads and/or Implementation Team Members to implement the innovation.	Who else is needed to support implementation, relative to the context and features of the nutrition innovation (e.g., administrative or IT staff, logistics support, graphic design, etc)?
**Innovation deliverers**	Individuals who are directly or indirectly delivering the innovation.	Who is responsible for directly delivering the nutrition innovation to the innovation recipient (e.g., dietitians, nurses, physicians), and who supports its delivery (e.g., administrators, IT staff, healthcare professionals)? How can the roles of the deliverers serve as barriers or facilitators to implementation?
**Innovation recipients**	Individuals who are directly or indirectly receiving the innovation.	Who are the target recipients of the nutrition innovation (e.g., patients, clients, caregivers)? How can the recipient serve as a barrier or facilitator to implementation?
**Characteristics subdomain** Project characteristics: the characteristics of individuals involved with implementing, *delivering, and/or receiving the innovation.*		
**Need**	The individual(s) has deficits related to survival, well‐being, or personal fulfillment, which will be addressed by implementation and/or delivery of the innovation.	Have the individual needs, wishes, and desires of the people implementing, delivering, or receiving the nutrition innovation been considered? Do the individuals involved perceive a significant need for the nutrition innovation?
**Capability**	The individual(s) has interpersonal competence, knowledge, and skills to fulfill Role.	Do the individuals involved with implementing, delivering, or receiving the nutrition innovation have the necessary skills and knowledge to feel confident and capable in fulfilling their roles? What additional training or resources are needed to enhance capability in healthy eating?
**Opportunity**	The individual(s) has availability, scope, and power to fulfill Role.	What factors outside the individual implementing, delivering or receiving the nutrition innovation make fulfilling their role possible (e.g., time, authority, staff availability, scheduling, autonomy, control, etc)? Which of these factors might serve as barriers to implementation and which can be leveraged?
**Motivation**	The individual(s) is committed to fulfilling Role.	Are the individuals responsible for implementing, delivering, or receiving the personally motivated and committed to engage with the nutrition innovation (intrinsic motivation)? What extrinsic factors outside the individual can influence motivation and commitment, relative to the nutrition innovation?

Abbreviation: CFIR, Consolidated Framework of Implementation Research.

* Description from: Damschroder et al.,^9^ licensed under CC BY 4.0 (https://creativecommons.org/licenses/by/4.0/).

### Inpatient case

After reflecting on the inner setting, the dietitian begins to characterize the structural roles of different individuals that will influence and support implementation. The dietitian has identified support for CNST implementation from meeting with hospital executives (high‐level leaders). However, successful implementation will also require engagement and communication with unit managers and managers of professional practice as well (midlevel leaders). Although nurses will be the primary persons to use the CNST on patient admissions (intervention deliverers), the dietitian also identifies physicians as being strong opinion leaders whose engagement and advocacy for malnutrition detection highly influences the teams’ enthusiasm for this initiative.

Building on the success of implementing the CNST in the surgical unit, the dietitian prepares for expansion by forming an institutional malnutrition task force and co‐chairs the committee with a nursing colleague (implementation leads). The task force includes interdisciplinary involvement from physicians, dietitians, nurse educators and other allied health staff (implementation team members). The mandate of this committee is to oversee implementation and sustainability of nutrition screening, assessment, and treatment of malnutrition. As part of the CNST initiative, the committee is tasked with supporting expanded implementation by identifying barriers and facilitators from their unique perspectives.

During the pilot implementation of the CNST in the surgical unit, the greatest challenge was engaging and encouraging nurses to complete the screening tool. In the postimplementation phase, the dietitian conducted a survey among the surgical unit nurses to obtain a better understanding of their views, guided by the CFIR, including questions on (1) their degree of self‐efficacy when it comes to using CNST (capability), (2) their experiences working with malnourished patients and their motivation to address it (need), (3) their enthusiasm and belief that malnutrition should be addressed and the perception of whether screening benefits patients (motivation), and (4) the support they feel is needed to sustain use of the CNST over the long‐term (opportunity). The results of this survey showed that surgical unit nurses, as innovation deliverers, found the CNST use to be associated with the following barriers: lack of time, concern over the length of admission assessments and perceived added workload (opportunity). However, reported facilitators in using the CNST include belief that it is quick and easy to use (capability), that the screening led to important and positive outcomes for patients (needs), and that the involvement of peer champions (implementation facilitators) helped to address their questions (capability) and provided a sense of extrinsic motivation to continue using it (motivation).

### Outpatient case

Recognizing the critical influence of individuals on implementation success, the dietitian systematically identifies the people and their respective roles within the clinic, examining how those involved will contribute to the delivery and adoption of weight‐inclusive care. A key driver for the implementation of weight‐inclusive care is the executive director's strong commitment to equity and accessibility, including the support of a weight‐neutral clinic space as a way to redress weight stigma (high‐level leaders). The dietitian, in this case, acts as both an implementation lead and innovation deliverer, championing advocacy, education, and integration of weight‐inclusive care into practice.

The dietitian possesses the authority and autonomy to deliver care within her role and scope of practice (opportunity); however her undergraduate and dietetic internship provided no formal training on using weight‐inclusive approaches in practice. On the dietitian's self‐assessment, it is recognized that their knowledge and skills regarding intuitive, trauma‐informed counseling, the social determinants of health, and frameworks for health equity, antioppression, and fat liberation are currently limited (capabilities). The dietitian will require training and mentorship to build the necessary competence. Although the dietitian is largely motivated by her clients (innovation recipients) and their frustration with weight‐focused dietary interventions (need), she recognizes, through a review of the literature, that some patients’ expectations of traditional weight‐loss counseling may serve as a significant barrier to the implementation of weight‐inclusive care in outpatient practice, reinforced by cultural norms.^43^

Nurses and physicians at the clinic would also play an indirect role as innovation deliverers, since one of the goals of adopting a weight‐inclusive approach is to ensure consistent messaging across the healthcare team within the clinic. However, to integrate weight‐inclusive care as part of clinical practices, the dietitian recognizes that physicians, as influential midlevel leaders in the clinic setting, may be more difficult to engage and pose as a barrier. Based on her informal conversations, the dietitian understands that some clinic physicians place high emphasis on weight loss as a primary treatment goal. As a result, the desire to uphold weight‐centric norms may be high among some physicians, limiting their intrinsic motivation and commitment to the weight‐inclusive approach and representing a significant implementation challenge (need, motivation). In contrast, the dietitian has learned that two clinic nurses are enthusiastic about the weight‐inclusive approach and are open to playing a key role in endorsing the approach and influencing team attitudes (implementation facilitators). Additionally, one supportive, charismatic physician leader who also serves on the clinic executive committee, has taken great interest in weight‐inclusive care and has committed to work to influence their peers in its adoption and implementation (opinion leader).

## IMPLEMENTATION PROCESS DOMAIN

The implementation process domain of the CFIR captures the core activities and strategies used to effectively roll‐out and integrate the innovation within a specific setting (Table 5). A tenant of this domain is that it recognizes that implementation is a dynamic and iterative process, not a single event, that unfolds over time and requires continuous monitoring and adaptation (evaluation). The activities within this domain can be formally conceptualized, planned, and enacted, or they can emerge informally or organically during the implementation process; however, all activities contribute to the overall success of implementation. Importantly, implementation activities and processes can be refined and reevaluated in an iterative fashion throughout the implementation process. Monitoring the implementation process is important as it can provide an understanding of the stages of implementation that a nutrition innovation has to undergo and the actions that are needed to occur at each stage for successful implementation. The key components of this domain include planning a clear, coordinated strategy to guide implementation considering identified barriers and enablers; engaging stakeholders (e.g., leaders, innovation deliverers, champions, innovation recipients) to maximize buy‐in and support for implementation; executing the implementation plan; and evaluating the implementation process over time. Evaluation efforts should focus on identifying what is working, where barriers exist, what enablers can be leveraged, and how improvements can be made to maximize success with implementation outcomes. By considering these elements, implementation teams can ensure their efforts are intentional, responsive, and enable longer‐term adoption, implementation, and sustainability. A well‐designed and well‐executed implementation process is essential for effectively translating a nutrition innovation into nutrition practice.

**Table 5:** CFIR implementation process domain constructs.

Construct	Description* The degree to which:	Example implementation questions related to nutrition innovations
**Teaming**	Join together, intentionally coordinating and collaborating on interdependent tasks, to implement the innovation.	To what extent are the people involved with implementing, delivering or receiving the nutrition innovation working together to implement the nutrition innovation? How are implementation activities coordinated and interconnected across these individuals?
**Assessing needs**	Collect information about priorities, preferences, and needs of people. *Use this construct to capture themes related to Assessing Needs that are not included in the subconstructs below*.	
A. Innovation deliverers	Collect information about the priorities, preferences, and needs of deliverers to guide implementation and delivery of the innovation.	Do innovation deliverers (e.g., clinicians, registered dietitians) have a preferred method for implementing the nutrition innovation? Do preferences vary by innovation deliverer? What aspects of the nutrition innovation do deliverers prioritize, such as time efficiency, cultural relevance of materials, or alignment with their existing workload and responsibilities?
B. Innovation recipients	Collect information about the priorities, preferences, and needs of recipients to guide implementation and delivery of the innovation.	Do innovation recipients (e.g., patients, caregivers) have any preferences or needs regarding how they are exposed to, or interact with, the nutrition innovation (e.g., in‐person or online, frequency of interactions)?
**Assessing context**	Collect information to identify and appraise barriers and facilitators to implementation and delivery of the innovation.	What data collection practices are in place to collect information on barriers and facilitators to adoption and delivery of the innovation intervention? What mechanisms are in place to monitor how the nutrition innovation is being received by the staff and stakeholders during implementation?
**Planning**	Identify roles and responsibilities, outline specific steps and milestones, and define goals and measures for implementation success in advance.	Have clearly defined roles, responsibilities, and timelines been established for planning and rolling out the nutrition innovation, and do these reflect the capacity and opportunities available to the individuals involved? Have specific and measurable implementation goals been established related to the nutrition innovation, such as the percentage of staff who adopted the innovation, the fidelity with implementation intervention procedures, or the number of staff who sustained their use of the intervention over the long‐term?
**Tailoring strategies**	Choose and operationalize implementation strategies to address barriers, leverage facilitators, and fit context.	Considering the context, how can implementation strategies be customized to minimize the barriers, and leverage the facilitators, to implementing the nutrition innovation?
**Engaging**	Attract and encourage participation in implementation and/or the innovation. *Use this construct to capture themes related to Engaging that are not included in the subconstructs below*.	
A. Innovation deliverers	Attract and encourage deliverers to serve on the implementation team and/or to deliver the innovation.	Have potential deliverers been encouraged to join the implementation team and participate in the development of the implementation plan? What incentives or recognition are being used to keep them engaged?
B. Innovation recipients	Attract and encourage recipients to serve on the implementation team and/or participate in the innovation.	Have potential recipients, who have a similar background to actual recipients, been encouraged to join the implementation team and participate in the development of the implementation plan? What incentives or recognition are being used to keep them engaged?
**Doing**	Implement in small steps, tests, or cycles of change to trial and cumulatively optimize delivery of the innovation.	Can implementation of the nutrition innovation take place gradually, in incremental steps (e.g., in a single unit, classroom or clinic), to make the implementation more manageable, to give deliverers time to learn and adapt, and to give the implementation team an opportunity to adjust the implementation plan as needed?
**Reflecting & evaluating**	Collect and discuss quantitative and qualitative information about the success of implementation. *Use this construct to capture themes related to Reflecting & Evaluating that are not included in the subconstructs below*.	
A. Implementation	Collect and discuss quantitative and qualitative information about the success of implementation.	What plans do the team have to collect and reflect on qualitative and quantitative data regarding how the nutrition innovation was implemented? How are these findings being used to guide improvement? To what extent were key implementation goals or milestones achieved and what factors influenced success or failure?
B. Innovation	Collect and discuss quantitative and qualitative information about the success of the innovation.	What plans does the team have to collect quantitative and/or qualitative data on outcomes that occurred as the result of the implementation of the nutrition innovation, such as recipient nutrition status, dietary knowledge, attitudes and behaviors, or health indices (eg blood cholesterol, blood pressure, hemoglobin A1c, quality of life)? How are the nutrition innovation deliverers, recipients, and decision makers describing the benefits or limitations of the nutrition innovation?
**Adapting**	Modify the innovation and/or the Inner Setting for optimal fit and integration into work processes.	What modifications have been made to either the nutrition innovation or to organizational workflows to improve fit, integration, or sustainability of the nutrition innovation? How are adaptations documented and communicated to innovation deliverers or recipients? Have adaptations improved implementation or innovation outcomes?

Abbreviation: CFIR, Consolidated Framework of Implementation Research.

* Description from: Damschroder et al.,^9^ licensed under CC BY 4.0 (https://creativecommons.org/licenses/by/4.0/).

### Inpatient case

In preparation for expanding the CNST to additional hospital units, the institutional malnutrition task force, co‐chaired by the dietitian, established a CNST Implementation Subcommittee to oversee the implementation process (teaming). This team includes nurse representatives from each of the hospital units in which the CNST will be implemented, who will speak to the unique needs and contexts of their unit and later serve as innovation champions (assessing needs: innovation deliverers; assessing context, engaging: innovation deliverers).

The subcommittee reflects on successes and barriers from the surgical unit pilot to create a plan that maximizes sustainable implementation into other hospital units (planning). To build on the findings from the postimplementation evaluation in the surgical unit, the subcommittee designs a preimplementation survey guided by the CFIR. The survey is conducted on hospital units in which CNST had not yet been implemented to gain an understanding of nurse's needs (the innovation deliverer) in other contexts (assessing context). Through these surveys, the subcommittee found that the barriers to implementation included an underestimation of malnutrition prevalence among nurses, a perception that lack of time may hinder screening, and a concern over added workload. However, facilitators include addressing malnutrition as highly valued for patient outcomes, an openness to play a part in reducing malnutrition, and an interest in more nutrition education.

After considering all of the information gleaned from an assessment of the CFIR constructs and surveys characterizing innovation deliverers, the implementation team plans strategies to modify and maximize success of CNST implementation on other units (planning, tailoring strategies). It was felt that implementation should feature education on the prevalence of malnutrition, its impact on patients, and how nurses can play a role in combating malnutrition (engaging: innovation deliverers). The committee works together to design training for nurses that considers the constructs in the characteristics subdomain (tailoring strategies). This includes a focus on how to use the tool quickly and accurately, on the prevalence of malnutrition, on the importance of malnutrition detection, and on how nurses play an important role in the process of identifying malnutrition. Implementation will also identify nursing champions to answer questions and encourage their colleagues to play a part in this initiative (engaging: innovation deliverers).

The dietitian and task force recognize that expansion of malnutrition screening to additional units will be a dynamic process, requiring goal setting, data collection, and follow‐up surveys to monitor progress *(*reflecting and evaluating: implementation). Using this information, the team schedules regular meetings to follow up on implementation success and opportunities to modify the implementation plan to better suit the needs of deliverers and recipients (adapting). The subcommittee will periodically collect data on metrics of success (e.g., percentage of patients screened) and surveys of staff to identify and appraise barriers and facilitators as part of the dynamic implementation process, with necessary iterative adaptations to follow (adapting).

### Outpatient case

After a thorough assessment of the constructs from the other CFIR domains, the dietitian has identified many contextual factors that may act as barriers or facilitators to implementation success (assessing context) and takes the initial steps toward implementation. First, the dietitian secures a commitment from the clinic's executive director to integrate the weight‐inclusive care principles within other weight‐neutral clinic initiatives (tailoring strategies). Second, as part of the implementation plan, the dietitian schedules training and mentorship to enhance skills in weight‐inclusive care prior to implementation and broader team engagement (planning). Finally, the dietitian forms and chairs an implementation advisory committee (teaming) that includes a patient representative (engaging: innovation recipient), a nurse, a physician and a social worker (engaging: innovation deliverers). Their initial steps are to determine what is needed for clinic staff to successfully transition to weight‐neutral care (assessing needs: innovation deliverers). These needs will be assessed through two separate roundtable discussions, with discussion questions guided by the CFIR: one with physicians and nurses and the other with patients and caregivers led by the dietitian and supported by advisory committee members (assessing context, assessing needs: innovation recipients; assessing needs: innovation deliverers*)*. Later, the advisory committee uses the data generated from the needs and context assessments to create goals, milestones, and a timeline for implementation (planning), including generating a series of strategies to address barriers to implementation (tailoring strategies).

The advisory committee will engage clinic staff. Internally, it will be important to assess the needs and secure the buy‐in of any physicians, nurses, administrative staff who may initially be skeptical or unaware of weight‐inclusive care (engaging: intervention deliverers). Since lack of time is a documented barrier that primary care physicians face,^44^ the team will leverage existing clinic structures to inform clinicians of the changes and use prescheduled meetings and workshops to feasibly deliver educational sessions focused on emerging evidence and skill building related to weight‐inclusive care (engaging: intervention deliverers). These opportunities will increase knowledge and promote awareness and acceptance of weight‐inclusive care, with a goal of minimizing tension and resistance to change.

After assessing and engaging the needs of staff, the advisory committee begins the many tasks required for the transition, such as updating intake forms and revising assessment tools (adapting). Within the electronic medical record system, mandatory intake fields for weight and weight‐loss goals are removed and fields for weight‐neutral assessments of health, such as eating behaviors and quality‐of‐life indicators, are added (adapting, tailoring strategies). The advisory committee then begins to identify the needs of patients as part of this transition (assessing needs: innovation recipients). Patient‐facing materials and resources, such as waiting room posters, pamphlets, and digital content, will require updating with weight‐neutral/weight‐inclusive language *and images (adapting, tailoring strategies*). Informing patients in advance of the clinic's shift to a weight‐inclusive approach will help manage expectations, reduce confusion, and foster trust (engaging: innovation recipients).

Finally, the implementation team will commit to reflecting and evaluating throughout the implementation process (reflecting and evaluating: implementation). This includes gathering feedback from patients and staff, monitoring shifts in clinical outcomes and patient and clinician satisfaction collected through anonymous surveys and roundtable discussions. Results will inform success of the changes and suggest changes required to ensure weight‐neutral care meets the needs of clients (adapting). Iterative learning and adaptation will be essential for the sustainable integration of weight‐inclusive care into the primary care clinic.

## TAKEAWAYS FROM THE CASE STUDIES

These case studies serve as a guide for teams on how to apply the CFIR framework to effectively implement and sustain nutrition innovations in real‐world practice settings. Specifically, the cases demonstrate how the CFIR can be applied in diverse nutrition contexts (i.e., malnutrition screening, dietary counseling) and settings (inpatient and outpatient) to identify the determinants that can influence implementation outcomes of nutrition innovations. By assessing factors that influence implementation across the five CFIR domains, we can proactively identify and address implementation challenges to optimize crucial implementation outcomes, including adoption, implementation, and sustainability. These are steps real‐world dietitians and clinicians can take to ensure successful and sustained utilization and impact of nutrition innovations to improve patient outcomes. Indeed, each of these cases could integrate formal and rigorous metrics for evaluation to better understand adoption, implementation, sustainability, and innovation effectiveness using established evaluation frameworks such as RE‐AIM.^13^

## STRENGTHS AND LIMITATIONS OF THE CFIR

A marked strength of the CFIR is that it draws on a wide range of theories from multiple disciplines to provide a unified structure for guiding implementation efforts. Beyond this, the CFIR offers additional advantages that contribute to its broad and practical application. First, the CFIR provides a shared language and a comprehensive and standardized list of constructs that can be used to guide researchers and practitioners in a detailed, systematic evaluation of determinants of implementation across diverse settings. The CFIR is highly adaptable and has been used in clinical, community, and organizational settings across various disciplines, including education, healthcare, and policy. This allows the CFIR to be used in the field of nutrition and dietetics to support implementation of nutrition innovations, programs, and policy. Moreover, the CFIR can be used in qualitative and quantitative research, to inform data collection tools (e.g., interview guides, surveys) and guide data analysis and interpretations of implementation findings. Additionally, the CFIR can be applied at different stages of implementation (i.e., before, during, and after implementation) to plan, monitor, and evaluate implementation strategies to support intervention delivery.^45^ The framework also supports adaptation of interventions and implementation strategies to address context‐specific needs to enhance adoption and successful intervention implementation.^45^ This is useful in the implementation of nutrition interventions, which often need to be adapted based on dietary preferences, culture, and availability of foods.

Despite the various strengths of the CFIR, there are also some limitations that researchers and practitioners should consider when applying the framework within nutrition and dietetic contexts. The major limitation of the CFIR lies in its comprehensiveness, which creates a level of complexity due to the number of constructs and the difficulty of discriminating between the relative importance of each construct and its influence on implementation outcomes. This is important in the context of nutrition innovations and interventions, which are often complex and involve multiple components and contexts, for example, access to healthy foods, food literacy skills, dietary behaviors, the social determinants of health, and interdisciplinary care teams, that influence implementation success at various levels. Additionally, the CFIR is a determinant framework and though it provides recommendations on outcomes to consider during implementation evaluation, it does not directly evaluate the implementation process. Therefore, it must be used in conjunction with other process evaluation frameworks, such as the RE‐AIM framework, that help to assess both implementation process and outcomes, examining factors such as reach, adoption, and fidelity. Moreover, although the CFIR identifies both people and context as key players in implementation, it does not address how these two interact overtime during the implementation process, a limitation that has been acknowledged by its creator.^7^, ^46^ This is due to the lack of implementation science research exploring the dynamic relationship between individuals and their organizational setting throughout implementation.^46^ This is particularly relevant in nutrition, in which individuals’ dietary behaviors change as a result of factors such as their food environment, habits, and access to resources. These factors also impact how nutrition interventions are delivered by providers; thus, a nuanced understanding of this evolving interaction is necessary for the successful design and implementation of nutrition interventions.

## CONCLUSIONS

This article presents a review of the CFIR and the application of the framework in the context of nutrition through two illustrative case studies. We demonstrated how the CFIR can be used to assess the barriers and facilitators to implementation of the CNST tool for malnutrition screening in an inpatient surgical unit and the weight‐inclusive approach to weight‐loss counseling in an outpatient primary care setting. The CFIR provides a unified framework of determinants of implementation and has strong potential for application in the field of nutrition and dietetics to guide the implementation of nutrition innovations to improve delivery of care and patient outcomes. There is a need for future research to map the full extent of CFIR's application in nutrition and dietetics, ideally through a scoping or systematic review. At the practice and policy level, clinicians and healthcare providers need to be trained on how to apply the CFIR framework in practice settings to support the effective implementation and integration of evidence‐based nutrition innovations. Moreover, embedding the CFIR into standardized program evaluations may promote more rigorous assessment, ultimately enhancing the delivery and impact of nutrition care.

## AUTHOR CONTRIBUTIONS

Bridve Sivakumar and JoAnne Arcand designed the scope and concept for the manuscript. All authors participated in the analysis of the literature, drafting of the paper and critically reviewing the draft paper for intellectual content. All authors approved the final version of the paper.

## CONFLICT OF INTEREST STATEMENT

The authors declare no conflicts of interest.
